# A Low-Complexity Algorithm for a Reinforcement Learning-Based Channel Estimator for MIMO Systems

**DOI:** 10.3390/s22124379

**Published:** 2022-06-09

**Authors:** Tae-Kyoung Kim, Moonsik Min

**Affiliations:** 1 Department of Electronic Engineering, Gachon University, Seongnam 13120, Korea; tk415kim@gmail.com; 2 School of Electronics Engineering, Kyungpook National University, Daegu 41566, Korea; 3 School of Electronic and Electrical Engineering, Kyungpook National University, Daegu 41566, Korea

**Keywords:** multiple-input multiple-output, channel estimation, Markov decision process, reinforcement learning

## Abstract

This paper proposes a low-complexity algorithm for a reinforcement learning-based channel estimator for multiple-input multiple-output systems. The proposed channel estimator utilizes detected symbols to reduce the channel estimation error. However, the detected data symbols may include errors at the receiver owing to the characteristics of the wireless channels. Thus, the detected data symbols are selectively used as additional pilot symbols. To this end, a Markov decision process (MDP) problem is defined to optimize the selection of the detected data symbols. Subsequently, a reinforcement learning algorithm is developed to solve the MDP problem with computational efficiency. The developed algorithm derives the optimal policy in a closed form by introducing backup samples and data subblocks, to reduce latency and complexity. Simulations are conducted, and the results show that the proposed channel estimator significantly reduces the minimum-mean square error of the channel estimates, thus improving the block error rate compared to the conventional channel estimation.

## 1. Introduction

Currently, multiple-input multiple-output (MIMO) is an essential technology in wireless communications [1,2,3,4,5,6]. Multiple antennas are easy to implement in wireless systems, and their use significantly increases system reliability and capacity. However, to utilize the advantages of multiple antennas, perfect channel information is required at both the transmitter and receiver. Meeting this necessity is generally impossible because of the characteristics of wireless channels.

Although perfect channel information is unavailable, many studies have been conducted to improve the accuracy of channel estimation [7,8,9,10,11,12,13,14,15,16,17,18,19,20,21]. These investigations were mostly based on the use of pilots whose information is shared by both the transmitter and receiver and employed least-squares and linear minimum-mean square-error (LMMSE) estimations [10,11,12]. This is because the two estimation methods reasonably perform with affordable complexities for wireless systems. However, their performance strongly depends on the number of pilots, which is generally limited in wireless systems because employing several pilots as resources degrades the spectral efficiency.

This limitation can be overcome using data in channel estimation, i.e., conducting data-aided channel estimation [13,14,15,16,17,18,19,20,21]. Its concept is to exploit a detected data symbol as an additional pilot. Because a detected data symbol may have an error, the accuracy of the channel estimation may be degraded by it. An iterative turbo approach is a good method to address this degradation because the improved detection performance achieved using an iterative turbo equalizer also increases the estimation accuracy of a channel [19,20,21,22,23,24,25]. However, the use of this iterative turbo approach is limited in wireless systems because of its inherent high complexity and latency.

Recently, a reinforcement learning (RL) approach was introduced in [26] for data-aided channel estimation. In this approach, a Markov decision process (MDP) problem is described to minimize the estimation error, and an RL algorithm is used to solve the MDP problem. Without an iterative approach, the RL solution resulted in a significant improvement compared to conventional channel estimations. However, this solution is difficult to implement in practical systems because of its considerable complexity and latency in computing the optimal policy. For example, using the approach in [26] to calculate the optimal policy requires all a posteriori probabilities (APPs) in a data block. In addition, its limitation is that the optimal policy is characterized by a specific discounting factor.

In this paper, a low-complexity channel estimator using an RL approach is proposed for MIMO systems. The key concept of this estimator is the selection of the detected data symbols obtained during data detection as additional pilot symbols. To achieve this, an MDP problem is first defined to minimize the channel estimation error where the Q-value function is generalized by a discounting factor. Subsequently, an RL solution is proposed that can be easily implement in wireless systems. To this end, concepts of backup samples and data subblocks are introduced, which significantly reduce the complexity and latency. The main contributions of this study are summarized as follows:

- A data-aided channel estimator is developed to optimize the selection of detected symbols for MIMO systems. An MDP problem is defined for this selection to minimize the mean-square-error (MSE) of the channel estimates. Compared with [26], a discounting factor is introduced in the Q-value function. The discounting factor adjusts the effects of rewards after the current state.
- A low-complexity RL algorithm is proposed. To achieve this efficiently, a data block is separated into multiple data subblocks and the optimal policy for the data subblocks is characterized. In the characterization, only partial soft information obtained from data detection is utilized to reduce the calculation latency. Unlike in [26], the optimal policy is calculated using only this partially obtained information; the remaining rewards are approximated under the assumption of perfect detection. Finally, the optimal policy is obtained using a closed-form expression. Note that the conventional RL algorithm in [26] can be employed after obtaining all soft information in a data block.
- The performance enhancement achieved for MIMO systems using the developed RL algorithm is evaluated. Simulations are conducted, and the results demonstrate that the proposed algorithm significantly reduces the performance degradation of conventional channel estimation. Based on the simulations, the proposed channel estimator using an approximate MDP presents a similar performance to that of the original MDP. In addition, the proposed channel estimator provides robustness in time-varying channels.

The remainder of this paper is organized as follows. Section 2 introduces a signal model including the channel estimation and data detection considered in this study. In Section 3, an MDP problem to select detected data symbols optimally to minimize the channel estimation error is defined. A low-complexity RL algorithm is proposed in Section 4. In Section 5, simulation results are discussed, to demonstrate the effectiveness of the developed algorithm. Finally, conclusions are presented in Section 6.

### Notation

Matrices $0_{m}$ and $I_{m}$ represent m×m all-zero and the m×m identity matrices, respectively. The superscripts ${\left(\cdot \right)}^{T}$ and ${\left(\cdot \right)}^{H}$ denote the transpose and the conjugate transpose, respectively. Operators E(·) and P(·) denote the expectation of a random variable and the probability of an event, respectively. Operators |·| and ${∥\cdot ∥}^{2}$ denote the cardinality of a set and the norm, respectively. Operators ${\left(\cdot \right)}^{-1}$, Tr(·), and CN denote the inverse, trace, and complex normal distribution, respectively. Set C represents a set of complex numbers.

## 2. Signal Model

This section describes the signal model for a MIMO system. Based on the signal model, the channel estimator and data detector considered in this study are introduced.

### 2.1. Signal Model

**Figure 1:**
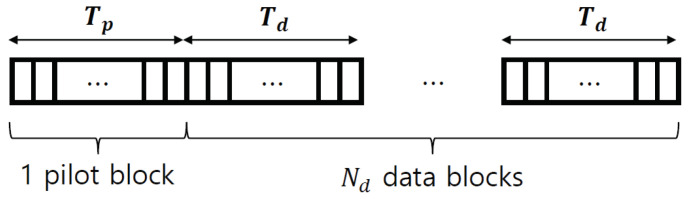
Frame consisting of one pilot block with $T_{p}$ symbols and $N_{d}$ data blocks with $T_{d}$ symbols.

A MIMO system is considered; in it, a transmitter with $N_{t}$ antennas communicates with a receiver with $N_{r}$ antennas through a wireless channel. A wireless channel is denoted as $H\in C^{N_{t}\times N_{r}}$, where each channel element $h_{t,r}\in C$ between the *t*-th transmitter and *r*-th receiver is modeled by Rayleigh fading $h_{t,r}∼\mathrm{CN}\left(0,1\right)$. The transmitter sends a frame consisting of one pilot block and $N_{d}$ data blocks, as shown in Figure 1. During the pilot transmission, the transmitter sends a pilot symbol $x^{p}\left[n\right]\in C^{N_{t}\times 1}$ for $n\in N_{p}=\left\{1,\ldots ,T_{p}\right\}$, where $T_{p}$ is the pilot length. When the pilot symbol $x^{p}\left[n\right]$ is transmitted to the receiver, the received symbol $y^{p}\left[n\right]\in C^{N_{r}\times 1}$ at time slot *n* is given as

(1) $$\begin{array}{cc} y^{p}\left[n\right] & =H^{H}x^{p}\left[n\right]+z^{p}\left[n\right], \end{array}$$

where $z^{p}\left[n\right]$ is an additive white Gaussian noise (AWGN) at time slot *n* whose distribution follows $\mathrm{CN}\left(0_{N_{r}},N_{0}I_{N_{r}}\right)$. After the pilot transmission is completed, the transmitter sends a data symbol $x^{d}\left[n\right]\in C^{N_{t}\times 1}$ for $n\in N_{d}=\left\{\left(d-1\right)T_{d}+1,\ldots ,dT_{d}\right\}$, where $T_{d}$ is the data length. Supposing X is a constellation set, the data symbol $x^{d}\left[n\right]\in X^{N_{t}}$. After the data transmission, the received symbol $y^{d}\left[n\right]\in C^{N_{r}\times 1}$ is expressed as

(2) $$\begin{array}{cc} y^{d}\left[n\right] & =H^{H}x^{d}\left[n\right]+z^{d}\left[n\right], \end{array}$$

where $z^{d}\left[n\right]$ is also an AWGN at time slot *n*.

### 2.2. Channel Estimator and Data Detector

The LMMSE channel estimator is considered in this study because of its satisfactory performance with low complexity. Using the received symbol in (1), the LMMSE channel estimator, $W\in C^{N_{t}\times T_{p}}$, is expressed as follows:

(3) $$\begin{array}{cc} \hat{W} & =\underset{W}{\mathrm{argmin}}E\left[∥W{\left(y_{r}^{p}\right)}^{H}-h_{r}{∥}^{2}\right]={\left(X^{p}{\left(X^{p}\right)}^{H}+N_{0}I_{N_{t}}\right)}^{-1}X^{p}, \end{array}$$

where $y_{r}^{p}$ and $X^{p}$ are sets of the received and pilot symbols and are defined as $y_{r}^{p}=\left[y_{r}^{p}\left[1\right],\cdots ,y_{r}^{p}\left[T_{p}\right]\right]$ and $X^{p}=\left[x^{p}\left[1\right],\cdots ,x^{p}\left[T_{p}\right]\right]$, respectively. Using the channel estimator in (3), a channel estimate is expressed as

(4) $$\begin{array}{cc} {\hat{h}}_{r} & =\hat{W}{\left(y_{r}^{p}\right)}^{H}={\left(X^{p}{\left(X^{p}\right)}^{H}+N_{0}I_{N_{t}}\right)}^{-1}X^{p}{\left(y_{r}^{p}\right)}^{H}, \end{array}$$

where ${\hat{h}}_{r}$ is the *r*-th row of the channel estimate matrix $\hat{H}$.

A maximum a posteriori probability (MAP) data detector is considered in this study to ensure the optimal detection performance. The APP from the MAP data detector is computed as    

(5) $$\begin{array}{cc} \theta_{k}\left[n\right] & =P\left[x^{d}\left[n\right]=x_{k}|y^{d}\left[n\right]\right]=\frac{P\left[y^{d}\left[n\right]|x^{d}\left[n\right]=x_{k}\right]P\left[x^{d}\left[n\right]=x_{k}\right]}{\sum_{j\in K}P\left[y^{d}\left[n\right]|x^{d}\left[n\right]=x_{j}\right]P\left[x^{d}\left[n\right]=x_{j}\right]}, \end{array}$$

where $x_{k}\in X^{N_{t}}$ is the *k*-th possible symbol for $k\in K=\{1,\ldots ,|X{|}^{N_{t}}\}$. In (5), the apriori probability, $P\left[x^{d}\left[n\right]=x_{k}\right]$, is assumed to be equal for all possible symbols $x_{k}$ for k∈K, i.e., $P\left[x^{d}\left[n\right]=x_{k}\right]=\frac{1}{{\left|X\right|}^{N_{t}}}$. Concurrently, under the AWGN assumption, the likelihood probability $P\left[y^{d}\left[n\right]|x^{d}\left[n\right]=x_{k}\right]$ in (5) can be expressed as

(6) $$\begin{array}{cc} P\left[y^{d}\left[n\right]|x^{d}\left[n\right]=x_{k}\right]\; & =\frac{1}{{\left(\pi N_{0}\right)}^{N_{r}}}e^{-\frac{∥y^{d}\left[n\right]-{\hat{H}}^{H}x_{k}{∥}^{2}}{N_{0}}}. \end{array}$$

The MAP data detector detects the data symbol $\hat{x}\left[n\right]$ that has the best APP value at time slot *n*, and it is given by

(7) $$\begin{array}{cc} \hat{x}\left[n\right] & =\underset{x_{k}\in X^{N_{t}}}{\mathrm{argmax}}\theta_{k}\left[n\right]=\underset{x_{k}\in X^{N_{t}}}{\mathrm{argmax}}P\left[y^{d}\left[n\right]|x^{d}\left[n\right]=x_{k}\right]. \end{array}$$

Note that the accuracy of the detected symbol $\hat{x}\left[n\right]$ depends on the accuracy of the channel estimator, $\hat{H}$. However, the accuracy of the channel estimator cannot be ensured in practical systems where the pilot length, $T_{p}$, is limited. To address this limitation, this study focused on improving the accuracy of the channel estimator.

## 3. Optimization Problem

This section defines the optimization problem for the channel estimator proposed subsequently, which uses detected symbols to improve the MSE of the channel estimates. Subsequently, to solve the optimization problem, the MDP problem and the optimal policy are presented.

### 3.1. Optimization Problem

This study considers a channel estimator that uses the detected symbols in (7) as additional pilot symbols. However, the data detector may generate detection errors at the receiver. Consequently, the use of detected symbols with errors degrades the accuracy of the channel estimator. To overcome this problem, the detected symbols should be selectively exploited by the channel estimator.

Let $a\in {\left\{0,1\right\}}^{T_{d}}$ be the set of actions whose *n*-th component is the selection of a detected symbol of the *d*-th data block for $n\in N_{d}$. Specifically, when a=1, a detected symbol is used as an additional pilot symbol; otherwise, it is not used. By exploiting a, the LMMSE channel estimate in (4) can be updated as

(8) $$\begin{array}{cc} {\hat{h}}_{r}\left(a\right) & ={\left(X\left(a\right)X{\left(a\right)}^{H}+N_{0}I_{N_{t}}\right)}^{-1}X\left(a\right){\bar{y}}_{r}{\left(a\right)}^{H}, \end{array}$$

where ${\bar{y}}_{r}\left(a\right)=\left[y_{r}^{p},y_{r}^{d}\left[u_{1}\left(a\right)\right],\ldots ,y_{r}^{d}\left[u_{{∥a∥}_{0}}\left(a\right)\right]\right]$ and $X\left(a\right)=[X^{p},\hat{x}\left[u_{1}\left(a\right)\right],\ldots ,\hat{x}\left[u_{{∥a∥}_{0}}\left(a\right)\right]]$.

Here, $u_{i}\left(a\right)$ is the time slot index of the *i*-th nonzero element in a. Thus, the optimization problem that maximizes the accuracy of the proposed channel estimator can be expressed as

(9) $$\begin{array}{cc} a^{★} & =\underset{a\in {\left\{0,1\right\}}^{T_{d}}}{\mathrm{argmax}}E\{∥\hat{H}\left(a\right)-H{∥}^{2}\}. \end{array}$$

Solving the optimization problem in (9) is difficult. First, the distribution of $\hat{H}\left(a\right)$ requires information regarding the transmitted symbols. However, this information is generally unknown to a receiver. In addition, the number of candidates for actions a exponentially increases with data length $T_{d}$. Accordingly, an exhaustive search for these actions is impractical because of the unsatisfactory complexity and latency for the receiver.

### 3.2. Markov Decision Process

To efficiently solve the problem in (9), an MDP was formulated in [26] that sequentially selected detected symbols. In this formulation, a detected symbol is selected if the updated channel estimator reduces the estimation error.

Similar to [26], for this study, the state set of the MDP at time slot *n* is expressed as

(10) $$\begin{array}{cc} S_{n}=\{\left(X_{n},{\hat{X}}_{n},M_{n}\right)|\; & X_{n}=\left[X^{p},x_{k_{M_{n}\left(1\right)}},\cdots ,x_{k_{M_{n}\left(|M_{n}|\right)}}\right],k_{i}\in K,\\ \; & {\hat{X}}_{n}=\left[X^{p},\hat{x}\left[M_{n}\left(1\right)\right],\cdots ,\hat{x}\left[M_{n}\left(|M_{n}|\right)\right]\right],\\ \; & M_{n}\subset \left\{T_{p}+1,\cdots ,n-1\right\}\}, \end{array}$$

where $k_{n}$ denotes the transmitted symbol index at time slot *n*. Set $M_{n}$ represents the set of time slot indices of the data symbols to be utilized as additional pilot symbols. $M_{n}\left(i\right)$ is the *i*-th smallest element of $M_{n}$. Based on the above notations, the proposed channel estimate at state $S_{n}=\left(X_{n},{\hat{X}}_{n},M_{n}\right)\in S_{n}$ is expressed as

(11) $$\begin{array}{cc} {\hat{h}}_{r}\left(S_{n}\right) & ={\left({\hat{X}}_{n}{\hat{X}}_{n}^{H}+N_{0}I_{N_{t}}\right)}^{-1}{\hat{X}}_{n}{\bar{y}}_{r}^{H}\left(S_{n}\right), \end{array}$$

where ${\bar{y}}_{r}\left(S_{n}\right)=[y_{r}^{p},y_{r}^{d}\left[M_{n}\left(1\right)\right],\ldots ,y_{r}^{d}[M_{n}\left(\right|M_{n}\left|)]\right]$.

The action set of the MDP is expressed as A={0,1}. An action is defined as whether to utilize a current detected symbol as an additional pilot symbol. Specifically, when a=1∈A, the current detected symbol is used as an additional pilot symbol.

Based on the state and action sets, the state transition function of the MDP for a∈A and $S_{n}\in S_{n}$ is expressed as follows:

(12) $$\begin{array}{cc} T_{n+1}^{\left(a,j\right)}\left(S_{n}\right)\; & =P\left[U_{n+1}^{\left(a,j\right)}\left(S_{n}\right)|S_{n},a\right]=\left\{\begin{array}{cc} I\left[x^{d}\left[n\right]=x_{j}\right], & j\in J_{a},a=1,\\ 1, & j\in J_{a},a=0. \end{array}\right. \end{array}$$

where $J_{0}=\left\{0\right\}$ and $J_{1}=\left\{1,\ldots ,K\right\}$. State $U_{n+1}^{\left(a,j\right)}\left(S_{n}\right)\in S_{n+1}$ is the valid state from the current state $S_{n}=\left(X_{n},{\hat{X}}_{n},M_{n}\right)\in S_{n}$, and is expressed as

(13) $$\begin{array}{cc} U_{n+1}^{\left(a,j\right)}\left(S_{n}\right)\; & =\left\{\begin{array}{cc} \left(\left[X_{n},x_{j}\right],\left[{\hat{X}}_{n},\hat{x}\left[n\right]\right],\left[M_{n}\cup n\right]\right), & j\in J_{a},a=1,\\ \left(X_{n},{\hat{X}}_{n},M_{n}\right), & j\in J_{a},a=0. \end{array}\right. \end{array}$$

The reward function of the MDP is obtained by the MSE improvement between the channel estimates at the current state $S_{n}$ and the next state $S_{n+1}$. Thus, the reward function from $S_{n}\in S_{n}$ to $S_{n+1}\in S_{n+1}$ is defined as

(14) $$\begin{array}{cc} R\left(S_{n},S_{n+1}\right) & =E_{r}\left(S_{n}\right)-E_{r}\left(S_{n+1}\right), \end{array}$$

where $E_{r}\left(S_{n}\right)$ is the MSE of the channel estimate for the *r*-th receive antenna at state $S_{n}\in S_{n}$, which can be computed as

(15) $$\begin{array}{cc} E_{r}\left(S_{n}\right)\; & =E\left[∥{\hat{h}}_{r}\left(S_{n}\right)-h_{r}{∥}^{2}\right]=\mathrm{Tr}\left[C_{e}\left(S_{n}\right)\right], \end{array}$$

where the error covariance matrix $C_{e}\left(S_{n}\right)$ is defined as $E\{\left({\hat{h}}_{r}\left(S_{n}\right)-h_{r}\right){\left({\hat{h}}_{r}\left(S_{n}\right)-h_{r}\right)}^{H}\}$.

Here, $C_{e}\left(S_{n}\right)$ is independent of the receiver antenna index, *r*, because the channel and noise distributions are the same for different receive antenna indices. Thus, the reward function in (14) can be simplified as

(16) $$\begin{array}{cc} R\left(S_{n},S_{n+1}\right) & =\mathrm{Tr}\left[C_{e}\left(S_{n}\right)-C_{e}\left(S_{n+1}\right)\right]. \end{array}$$

The optimal policy of the MDP at time slot *n* is defined as

(17) $$\begin{array}{cc} \pi^{★}\left(S_{n}\right) & =\underset{a\in A}{\mathrm{argmax}}Q\left(S_{n},a\right). \end{array}$$

where the Q-value function $Q\left(S_{n},a\right)$ is the optimal sum of the rewards. Based on the state transition function in (12), the Q-value function can be expressed as

(18) $$\begin{array}{cc} Q\left(S_{n},a\right) & =\sum_{j\in J_{a}}T_{n+1}^{\left(a,j\right)}\left(S_{n}\right)\left[R\left(S_{n},U_{n+1}^{\left(a,j\right)}\left(S_{n}\right)\right)+\gamma V^{★}\left(U_{n+1}^{\left(a,j\right)}\left(S_{n}\right)\right)\right], \end{array}$$

where 0≤γ≤1 is a discounting factor whose value depends on the target of the optimization problem. For example, a small value is desirable when the accuracy of the channel estimator obtained at the current state is significant. In contrast, a larger value is preferred when the accuracy of the channel estimator obtained at the ending state is significant.

$V^{★}\left(U_{n+1}^{\left(a,j\right)}\left(S_{n}\right)\right)$ is the optimal sum of the future rewards. The future value function $V^{★}\left(S_{m}\right)$ at state $S_{m}\in S_{m}$ for n+1≤m can be recursively computed, as follows:

(19) $$\begin{array}{cc} V^{★}\left(S_{m}\right) & =\sum_{a\in A}\pi \left(S_{m},a\right)\sum_{j\in J_{a}}T_{m+1}^{\left(a,j\right)}\left(S_{m}\right)\left[R\left(S_{m},U_{m+1}^{\left(a,j\right)}\left(S_{m}\right)\right)+\gamma V^{★}\left(U_{m+1}^{\left(a,j\right)}\left(S_{m}\right)\right)\right], \end{array}$$

where $\pi \left(S_{m},a\right)$ is a state–action transition function, expressed as

(20) $$\begin{array}{cc} \pi \left(S_{m},a\right) & =I\{a=\underset{a^{\prime }\in A}{\mathrm{argmax}}Q\left(S_{m},a^{\prime }\right)\}, \end{array}$$

where $Q\left(S_{m},a\right)$ is the *Q*-value function that can be calculated as the sum of the rewards obtained after taking action a∈A at state $S_{m}\in S_{m}$.

**Figure 2:**
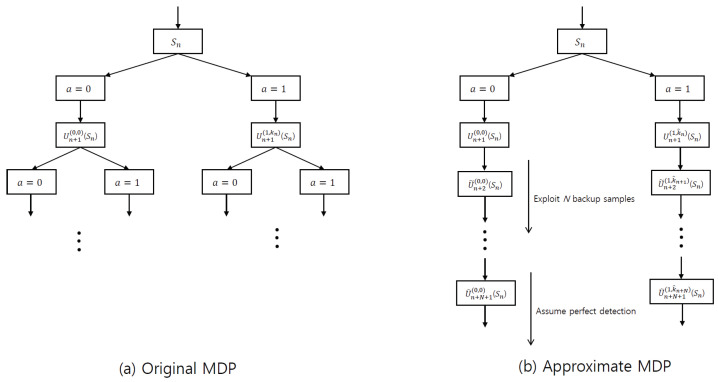
State–action diagrams of the original MDP (**a**) where $k_{n}$ is the transmitted symbol index, and the approximate MDP (**b**) where ${\hat{k}}_{n}$ is the detected symbol index for a∈A and $S_{n}\in S_{n}$.

Using the MDP in (10), (12), and (13), the state–action diagram of the original MDP is depicted in Figure 2a. In this figure, state $S_{n}$ is transited to the next valid state, $U_{n+1}^{\left(a,j\right)}\left(S_{n}\right)$, based on action *a*. Particularly, when a=1, state $S_{n}$ is transited to state $U_{n+1}^{\left(1,k_{n}\right)}\left(S_{n}\right)$ by utilizing the transmitted symbol index, $k_{n}$. Based on the state and state–action transition functions in (12) and (20), the state is transited to the next valid state until the end of a data block. As previously mentioned, the original MDP, which is shown in Figure 2a, cannot be solved by dynamic programming.

First, the state and state–action functions are unavailable to the receiver because the information of the transmitted symbols, $x_{k_{n}}$, and the true channel information, H, are unknown. In addition, the computational complexity and latency required to solve the original MDP are extremely high because the number of states exponentially increases with data length $T_{d}$.

## 4. Proposed Rl-Based Channel Estimator

In this section, an RL-based channel estimator is proposed. To address the unknown state and state–action functions, an RL algorithm is adopted because it provides a solution for the partially observable MDP [27,28]. Based on this algorithm, a computationally efficient RL solution is also proposed. The key concept of the proposed solution is to approximate the state–action transition functions to determine the optimal policy by separating the cases using the APPs.

**Figure 3:**
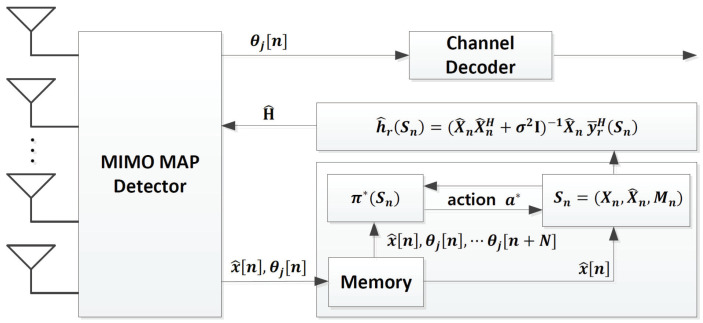
System structure of the proposed data-aided channel estimator.

The overall procedure of the proposed RL-based channel estimator is illustrated in Figure 3. The proposed channel estimator exploits the information of ($\hat{x}\left[m\right],\theta_{j}\left[m\right]$) obtained from the MIMO detector. In the proposed channel estimator, the optimal policy is calculated by using only N APPs $\left(\theta_{j}\left[n\right],\ldots ,\theta_{j}\left[n+N\right]\right)$ for a computationally efficient algorithm. The channel estimate is then updated according to the optimal policy. Details of the proposed channel estimator, i.e., how to approximate the MDP and how to derive the optimal policy in a closed form, are explained in this section.

### 4.1. Statistical State Transition

In this section, the state transition function in (12) at time slot *n* is approximated using the APP $\theta_{j}\left[n\right]$. The basic concept was introduced in [26] by assuming the APP $\theta_{j}\left[n\right]$ as the probability of the event, $\left\{x\left[n\right]=x_{j}\right\}$. Thus, the state transition function in (12) at time slot *n* is approximated as follows:

(21) $$\begin{array}{cc} {\hat{T}}_{n+1}^{\left(a,j\right)}\left(S_{n}\right)\; & =\left\{\begin{array}{cc} \theta_{j}\left[n\right], & j\in J_{a},a=1,\\ 1, & j\in J_{a},a=0. \end{array}\right. \end{array}$$

where the detected symbol index at time slot *n* is denoted as ${\hat{k}}_{n}$. Note that APP $\theta_{j}\left[n\right]$ can be interpreted as the probability of the event $\left\{x\left[n\right]=x_{j}\right\}$; thus, it is called a statistical transition. In addition, when the data detection performance is improved, i.e., $\theta_{k_{n}}\left[n\right]\to 1$, the approximate state transition function in (21) approaches the true state transition function in (12).

### 4.2. State–Action Transition Using Backup Samples

After time slot n+1≤m, the state in (20) is assumed to be transited to a virtual state that mimics the possible next states by exploiting the expected transmitted symbol, $\tilde{x}\left[m\right]$. The expected transmitted symbol, $\tilde{x}\left[m\right]$, is defined as

(22) $$\begin{array}{cc} \tilde{x}\left[m\right] & =\sum_{j=1}^{K}\theta_{j}\left[m\right]x_{j}. \end{array}$$

In this study, the use of the expected transmitted symbol is the same as in [26], except its use is limited to *N* backup samples to reduce the complexity. A backup sample is defined as APP $\theta_{j}\left[m\right]$ for n+1≤m≤n+N because the expected transmitted symbol can be computed by $\theta_{j}\left[m\right]$. Thus, the Q-value function can be calculated after all $\theta_{j}\left[m\right]$ for n+1≤m≤n+N values are obtained. Using a backup sample of an APP, the state–action transition is expressed as

(23) $$\begin{array}{cc} \hat{\pi }\left(S_{m},a\right) & =1. \end{array}$$

Thus, the virtual state, ${\tilde{U}}_{m}^{\left(a,j\right)}\left(S_{n}\right)\in S_{m}$, that can be transited from $S_{n}\in S_{n}$ is expressed as

(24) $$\begin{array}{cc} {\tilde{U}}_{m}^{\left(a,j\right)}\left(S_{n}\right)\; & =\left(X_{m}^{\left(a,j\right)},{\hat{X}}_{m}^{\left(a\right)},M_{m}^{\left(a\right)}\right), \end{array}$$

where their components are

$$\begin{array}{cc} X_{m}^{\left(a,j\right)} & =\left\{\begin{array}{cc} \left[X_{n},x_{j},\tilde{x}\left[n+1\right],\cdots ,\tilde{x}\left[n+N\right]\right], & a=1,\\ \left[X_{n},\tilde{x}\left[n+1\right],\cdots ,\tilde{x}\left[n+N\right]\right], & a=0. \end{array}\right.\\ {\hat{X}}_{m}^{\left(a\right)} & =\left\{\begin{array}{cc} \left[{\hat{X}}_{n},\hat{x}\left[n\right],\tilde{x}\left[n+1\right],\cdots ,\tilde{x}\left[n+N\right]\right], & a=1,\\ \left[{\hat{X}}_{n},\tilde{x}\left[n+1\right],\cdots ,\tilde{x}\left[n+N\right]\right], & a=0. \end{array}\right.\\ M_{m}^{\left(a\right)} & =\left\{\begin{array}{cc} \left[M_{n}\cup \left\{n,\ldots ,n+N\right\}\right], & a=1,\\ \left[M_{n}\cup \left\{n+1,\ldots ,n+N\right\}\right], & a=0. \end{array}\right. \end{array}$$

Because a virtual state mimics the transitions to the candidate symbols, state ${\tilde{U}}_{m}^{\left(a,j\right)}\left(S_{n}\right)\in S_{m}$ is always transited to a virtual state ${\tilde{U}}_{m+1}^{\left(a,j\right)}\left(S_{n}\right)\in S_{m+1}$. Therefore, the corresponding state transition function is written as

(25) $$\begin{array}{cc} {\hat{T}}_{m+1}^{\left(a,j\right)}\left({\tilde{U}}_{m}^{\left(a,j\right)}\left(S_{n}\right)\right) & =1, \end{array}$$

where n+1≤m≤n+N.

### 4.3. State–Action Transition after Backup Samples

In this subsection, the virtual states after n+N that can be transited without the information of the backup samples, $\theta_{j}\left[m\right]$, are described for n+N+1≤m. To achieve this, the states, ${\hat{U}}_{m+1}^{\left(a,j\right)}\left(S_{n}\right)$, for n+N+1≤m are assumed to optimally act when all symbols are correctly detected. By using the property of $x\left[m\right]=\hat{x}\left[m\right]$ after time slot n+N+1, an approximate virtual state is expressed as   

(26) $$\begin{array}{c} {\hat{U}}_{m}^{\left(a,j\right)}\left(S_{n}\right)=\left(X_{m}^{\left(a,j\right)},{\hat{X}}_{m}^{\left(a\right)},M_{m}^{\left(a\right)}\right), \end{array}$$

where its components are defined as

$$\begin{array}{cc} X_{m}^{\left(a,j\right)} & =\left[X_{n+N+1}^{\left(a,j\right)},\hat{x}\left[n+N+1\right],\cdots ,\hat{x}\left[m-1\right]\right],\\ {\hat{X}}_{m}^{\left(a\right)} & =\left[{\tilde{X}}_{n+N+1}^{\left(a\right)},\hat{x}\left[n+N+1\right],\cdots ,\hat{x}\left[m-1\right]\right],\\ M_{m}^{\left(a\right)} & =\left[M_{n+N+1}^{\left(a\right)}\cup \left\{n+N+1,\ldots ,m-1\right\}\right], \end{array}$$

where $\left(X_{n+N+1}^{\left(a,j\right)},{\hat{X}}_{n+N+1}^{\left(a\right)},M_{n+N+1}^{\left(a\right)}\right)$ are the components of ${\tilde{U}}_{n+N+1}^{\left(a,j\right)}\left(S_{n}\right)$.

In Figure 2b, a state–action diagram of the approximate MDP is depicted. The original MDP requires information regarding the transmitted symbols for the state transition, as shown in Figure 2a. In contrast, the approximate MDP utilizes virtual states ${\tilde{U}}_{m}^{\left(a,j\right)}\left(S_{n}\right)$ and ${\hat{U}}_{m}^{\left(a,j\right)}\left(S_{n}\right)$, which mimic the transitions to the candidate symbols for an unknown transmitted symbol and action. Specifically, virtual state ${\tilde{U}}_{m}^{\left(a,j\right)}\left(S_{n}\right)$ is used at time slot n+1≤m≤n+N and after time slot n+N, respectively. These two approximations decrease the number of transitions to the next state transition, so the calculation to solve the MDP is considerably reduced.

### 4.4. Proposed Optimal Policy

**Figure 4:**
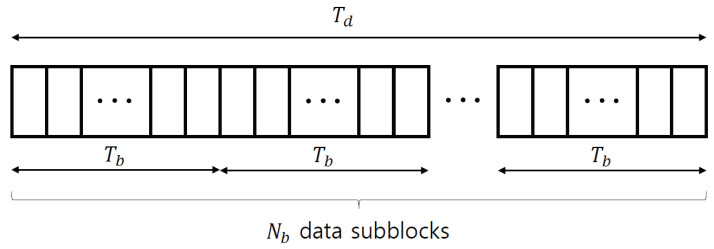
*d*-th data block consists of $N_{b}$ data subblocks with $T_{b}$ symbols.

Using the approximations in (21), (23), and (24), the optimal policy can be determined. However, the calculation latency is still considerable, because the optimal policy can be computed at the end of a data block. To prevent this computational burden, the proposed solution separates a data block into $N_{b}$ data subblocks and subsequently characterizes the optimal policy for each data subblock, as shown in Figure 4. Based on this characterization, the state in (10) and the corresponding channel estimate using (11) are updated for a data subblock. To realize this data subblock separation, the data subblock length is defined as $T_{b}$, which satisfies $N_{b}=T_{d}/T_{b}$. Thus, a set of time slot indices of the *b*-th data subblock in the *d*-th data block, $N_{b,d}$, is defined as $\left\{T_{p}+\left(b-1\right)T_{b}+\left(d-1\right)T_{d}+1,\ldots ,T_{p}+bT_{b}+\left(d-1\right)T_{d}\right\}$, for $b\in \{1,\ldots ,N_{b}\}$ and $d\in \{1,\ldots ,N_{d}\}$ (see Figure 4).

Using the virtual states in (24) and (26), the *Q*-value function is written as

(27) $$\begin{array}{cc} Q\left(S_{n},a\right) & =\sum_{j\in J_{a}}T_{n+1}^{\left(a,j\right)}\left(S_{n}\right)[R\left(S_{n},{\tilde{U}}_{n+1}^{\left(a,j\right)}\left(S_{n}\right)\right)+\sum_{m=n+1}^{n+N}\gamma^{m-n}R\left({\tilde{U}}_{m}^{\left(a,j\right)}\left(S_{n}\right),{\tilde{U}}_{m+1}^{\left(a,j\right)}\left(S_{n}\right)\right)\\ \; & +\gamma^{N+1}V^{★}\left({\hat{U}}_{n+N+1}^{\left(a,j\right)}\left(S_{n}\right)\right)], \end{array}$$

where the future value function, $V^{★}\left({\hat{U}}_{n+N+1}^{\left(a,j\right)}\left(S_{n}\right)\right)$, is obtained based on the approximation of ${\hat{U}}_{m}^{\left(a,j\right)}\left(S_{n}\right)$ as follows:   

(28) $$V^{★}\left({\hat{U}}_{n+N+1}^{\left(a,j\right)}\left(S_{n}\right)\right)\approx R\left({\tilde{U}}_{n+N+1}^{\left(a,j\right)}\left(S_{n}\right),{\hat{U}}_{n+N+2}^{\left(a,j\right)}\left(S_{n}\right)\right)+\sum_{m=n+N+2}^{N_{b,d}\left(T_{b}\right)}R\left({\hat{U}}_{m}^{\left(a,j\right)}\left(S_{n}\right),{\hat{U}}_{m+1}^{\left(a,j\right)}\left(S_{n}\right)\right).$$

In the future reward in (28), the discounting factor is assumed to be 1 to reduce the complexity by a simple calculation.

Based on (27) and (28), the optimal policy for each state is obtained as a closed-form expression, as described in the following theorem:

**Theorem** **1.** *Under the virtual states and the use of backup samples, the optimal policy for the state $S_{n}=\left(X_{n},{\hat{X}}_{n},M_{n}\right)\in S_{n}$ is*

(29) $$\begin{array}{cc} \pi^{★}\left(S_{n}\right) & =I\left[\frac{\sum_{m=n}^{n+N}\gamma^{m-n}\left(1-\gamma \right)U_{m}\left(S_{n}\right)+\gamma^{N+1}U_{N_{b,d}\left(T_{b}\right)+1}\left(S_{n}\right)}{\sum_{m=n}^{n+N}\gamma^{m-n}\left(1-\gamma \right)L_{m}\left(S_{n}\right)+\gamma^{N+1}L_{N_{b,d}\left(T_{b}\right)+1}\left(S_{n}\right)}\geq 1\right], \end{array}$$

*where functions $U_{m}\left(S_{n}\right)$ and $L_{m}\left(S_{n}\right)$ are respectively defined as*

$$\begin{array}{cc} U_{m}\left(S_{n}\right) & =∥t_{m}{∥}^{2}\left(N_{0}+N_{0}^{2}∥t_{m}{∥}^{2}+{∥v_{m}∥}^{2}\right)\\ L_{m}\left(S_{n}\right) & =∥t_{m}{∥}^{2}\left(2N_{0}^{2}\beta_{m}+\delta_{m}+{∥e_{m}-u_{m}+v_{m}∥}^{2}\right) \end{array}$$

*All components are defined as*

(30) $$\begin{array}{cc} \; & Q_{m}={\left({\hat{X}}_{n}{\hat{X}}_{n}^{H}+\sum_{l=n+1}^{m}\tilde{x}\left[l\right]{\tilde{x}}^{H}\left[l\right]+N_{0}I_{N_{t}}\right)}^{-1},\;D_{m}={\hat{X}}_{n}{\left({\hat{X}}_{n}-X_{n}\;\right)}^{H}+\sum_{l=n+1}^{m}\hat{x}\left[l\right]{\left(\hat{x}\left[l\right]-\tilde{x}\left[l\right]\right)}^{H}+N_{0}I_{N_{r}},\\ \; & t_{m}=\frac{1}{\sqrt{1+\alpha_{m}}}Q_{m}\hat{x}\left[n\right],\;e_{m}=\frac{1}{\sqrt{1+\alpha_{m}}}\left(\hat{x}\left[n\right]-\tilde{x}\left[n\right]\right),\;u_{m}=D_{m}^{H}t_{m},\;v_{m}=\frac{D_{m}^{H}Q_{m}t_{m}}{∥t_{m}{∥}^{2}},\\ \; & \alpha_{m}={\hat{x}}^{H}\left[n\right]Q_{m}\hat{x}\left[n\right],\;\beta_{m}=\frac{t_{m}^{H}Q_{m}t_{m}}{∥t_{m}{∥}^{2}},\;\delta_{m}=\frac{1}{1+\alpha_{m}}\left(\sum_{j=1}^{K}\theta_{j}\left[n\right]∥\hat{x}\left[n\right]-x_{j}{∥}^{2}-{∥\hat{x}\left[n\right]-\tilde{x}\left[n\right]∥}^{2}\right)\\ \; & Q_{N_{b,d}\left(T_{b}\right)+1}={\left(Q_{n+N}^{-1}+\left(N_{b,d}\left(T_{b}\right)-\left(n+N-1\right)\right)I_{N_{t}}\right)}^{-1},\;D_{N_{b,d}\left(T_{b}\right)+1}=D_{n+N}. \end{array}$$

**Proof.**  See Appendix A. □

### 4.5. Summary: The Proposed Algorithm

The proposed channel estimator is summarized in Algorithm 1. First, the receiver initializes the state during pilot transmission. In this algorithm, the current state is updated and transited to the next state according to the optimal action obtained using (29). For example, the most probable state transition is used when $\alpha^{★}=1$ for the unknown transmitted symbol index. This transition ensures a true state transition as $\theta_{j}\left[n\right]$ approaches 1 in reliable communication. At the end of a data subblock, the proposed channel estimator updates the channel estimate using the current state, $S_{n}$.  
**Algorithm 1:** The proposed channel estimator.  **1**  Set $H\leftarrow \hat{H}=\left[{\hat{h}}_{1},\cdots ,{\hat{h}}_{N_{r}}\right]$ from (4)  **2**  Initialize $S_{1}=\left(X^{p},X^{p},\phi \right)$.  **3**  **for** *d=1 to $N_{d}$***do**(  **4**   **for** *b=1 to $N_{b}$***do**  **5**    **for** $n\in N_{b,d}$**do**  **6**       Obtain $\hat{x}\left[n\right]$ from (8) and $\left\{\theta_{j}\left[n\right],\cdots ,\theta_{j}\left[n+N\right]\right\}$ from (5) for j∈K  **7**       Compute $a^{★}=\pi^{★}\left(S_{n}\right)$ from (29).  **8**       Set $j^{★}=0$ for $a^{★}=0$ and $x_{j^{★}}=\hat{x}\left[n\right]$ for $a^{★}=1$.  **9**       Update $S_{n+1}\leftarrow U_{n+1}^{\left(a^{★},j^{★}\right)}\left(S_{n}\right)$ from (13). **10**    **end** **11**    Set $H\leftarrow \hat{H}=\left[{\hat{h}}_{1}\left(S_{n}\right),\cdots ,{\hat{h}}_{N_{r}}\left(S_{n}\right)\right]$ from (11). **12**  **end** **13**  **end**

### 4.6. Complexity Analysis

In this subsection, the complexity of both the proposed channel estimator and that in [26] is discussed based on the number of states visited in the calculation of the optimal policy. This is because the rewards in the optimal policy are computed based on the states, and the calculation in (29) is similar to that in [26]. First, when the current state is $S_{n}\in S_{n}$ in the *d*-th data block, the number of visiting states in [26] is exactly $dT_{d}-n$. By contrast, the number of visiting states using the proposed channel estimator in the *b*-th data subblock is exactly $\left(b-1\right)T_{b}+1+\left(d-1\right)T_{d}-n$. Thus, the number of states ($T_{d}-\left(b-1\right)T_{b}-1$) is not used in the policy calculation on introducing the data subblocks. In addition to the complexity, the proposed optimal policy can be calculated after obtaining *N* backup samples, whereas in the approach in [26], this is possible at the end of a data block. Thus, the latency of the optimal policy by the approach in [26] is much longer than that of the proposed optimal policy.

## 5. Simulation Results

This section discusses the performance of the proposed channel estimator. The number of antennas in MIMO systems is $\left(N_{t},N_{r}\right)=\left(4,4\right)$. A rate 1/2 turbo code is adopted for channel coding, and 4-quadrature amplitude modulation (QAM) is adopted for symbol mapping. The frame consists of $\left(T_{p},T_{d},N_{d}\right)=\left(8,64,20\right)$, and the proposed channel estimator utilizes a data subblock as $\left(T_{b},N_{b}\right)=\left(16,4\right)$. In addition, the parameters of the proposed channel estimator are (N,γ)=(1,0.5), unless specified otherwise. The per-bit signal-to-noise ratio (SNR) is defined as $E_{b}/N_{0}=\frac{1}{\log_{2}\left|X\right|N_{0}}$.

**Figure 5:**
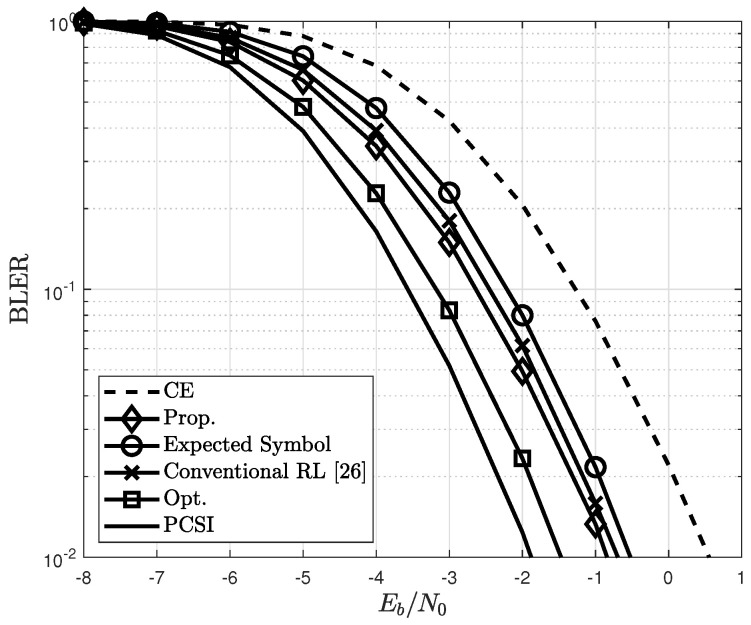
BLERs of conventional and proposed channel estimators for the different estimations.

In all figures, the performance with perfect and imperfect channel estimates using the LMMSE method are denoted as PCSI and CE, respectively. For performance benchmarking, the optimal cases of the proposed channel estimator and the expected-symbol-based channel estimator utilizing perfect knowledge of the transmitted symbol and the expected symbol in (22) as an additional pilot symbol, respectively, are compared. The performance is measured in terms of the block error rate (BLER) and the normalized MSE (NMSE). In Figure 5, the proposed channel estimator is compared with other channel estimators, and the conventional RL method used in [26] is also depicted. It shows that the BLER of the proposed estimator is better than those of the conventional and expected-symbol-based estimators regardless of the per-bit SNR. Moreover, the proposed channel estimator outperforms the conventional estimator of [26]. This is because the proposed channel estimator updates a channel estimate by $N_{b}$ in a data block, whereas the method in [26] updates it once at the end of a data block.

**Figure 6:**
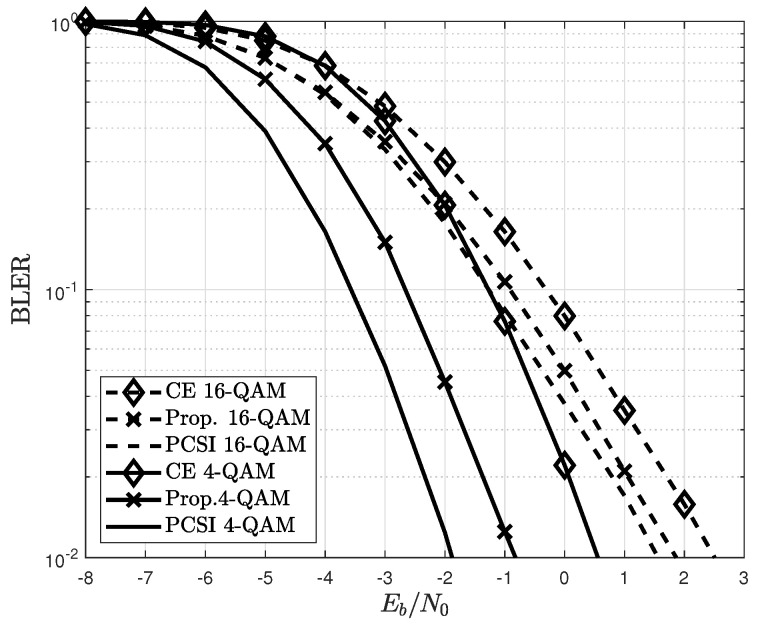
BLERs of conventional and proposed channel estimators for different modulations.

Figure 6 compares the BLERs of the conventional and proposed channel estimators for different modulations. For 16-QAM, a MIMO system with $\left(N_{t},N_{r}\right)=\left(2,4\right)$ is considered because of the SNR range. The proposed channel estimator achieves an improved BLER compared to the conventional LMMSE channel estimators. This result demonstrates the effectiveness of the proposed channel estimator, which optimizes the selection of detected symbols. The improvements to achieve a BLER of $10^{-1}$ are approximately 1.2 dB and 0.7 dB for the 4- and 16-QAM, respectively. The BLER for the 16-QAM is more improved than that of the PCSI, which is better than that of the 4-QAM. This is because in 16-QAM, the number of reliable detected symbols that can be used as additional pilot symbols is larger than in 4-QAM.

**Figure 7:**
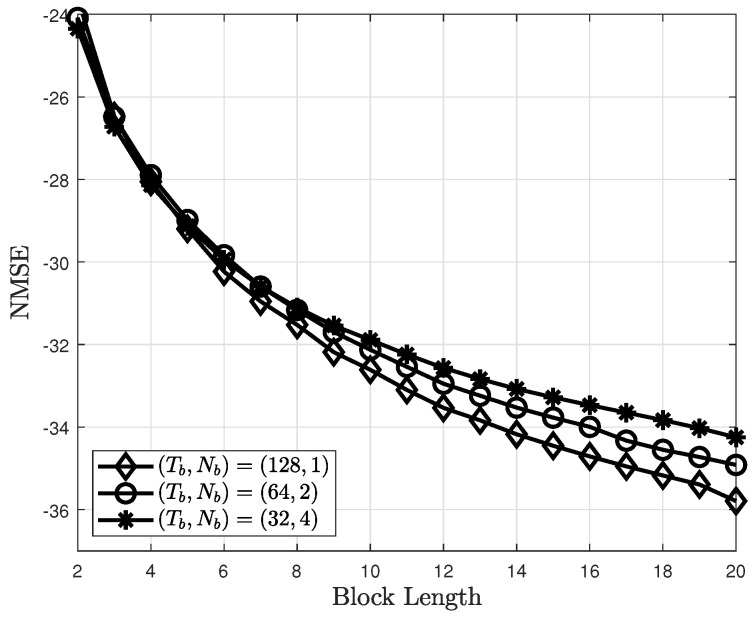
NMSEs of the proposed channel estimator for different $T_{b}$ and $N_{b}$.

The NMSEs of the proposed channel estimator for different data subblock lengths are shown in Figure 7. The NMSE improves as $N_{b}$ decreases. This is because the approximate MDP using data subblocks approaches the original MDP as $N_{b}$ decreases. However, as shown in Figure 7, the NMSE improvement is insignificant, whereas the complexity exponentially increases with $T_{b}$. Thus, $\left(T_{b},N_{b}\right)=\left(16,4\right)$ is considered in this study for the simulations.

**Figure 8:**
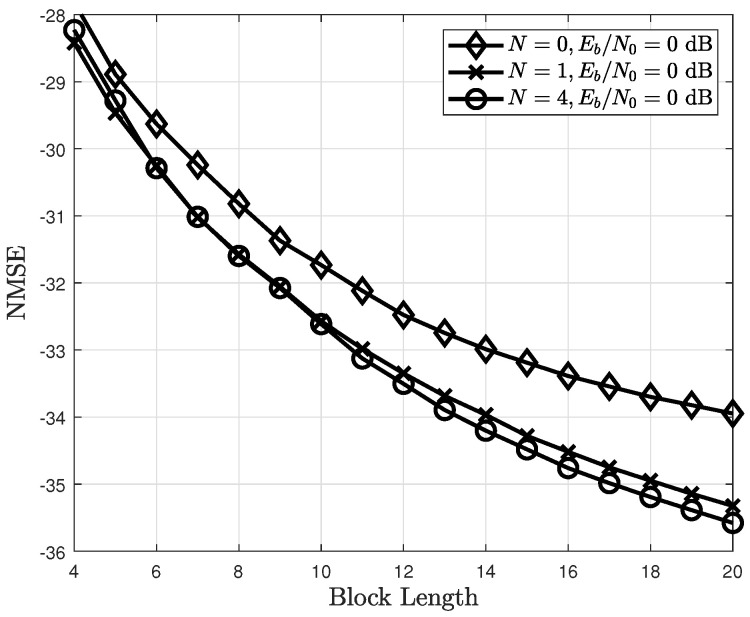
NMSE of the proposed channel estimator based on the number of backup samples *N*.

The NMSE of the proposed channel estimator based on the number of backup samples is shown in Figure 8. Noticeably, the NMSE is improved as the number of backup samples increases. This is because the accuracy of the state–action diagram model improves as the number of backup samples increases. In addition, with a small value of *N*, the proposed channel estimator achieves a sufficient NMSE performance. It should be noted that the complexity and latency required to determine the optimal policy increase with the number of backup samples.

**Figure 9:**
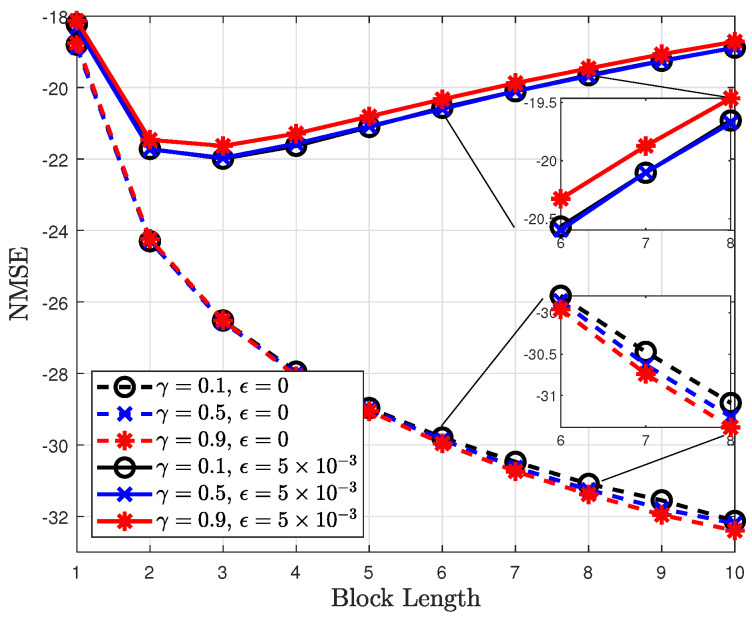
NMSEs of the proposed channel estimator for different discounting factors.

**Figure 10:**
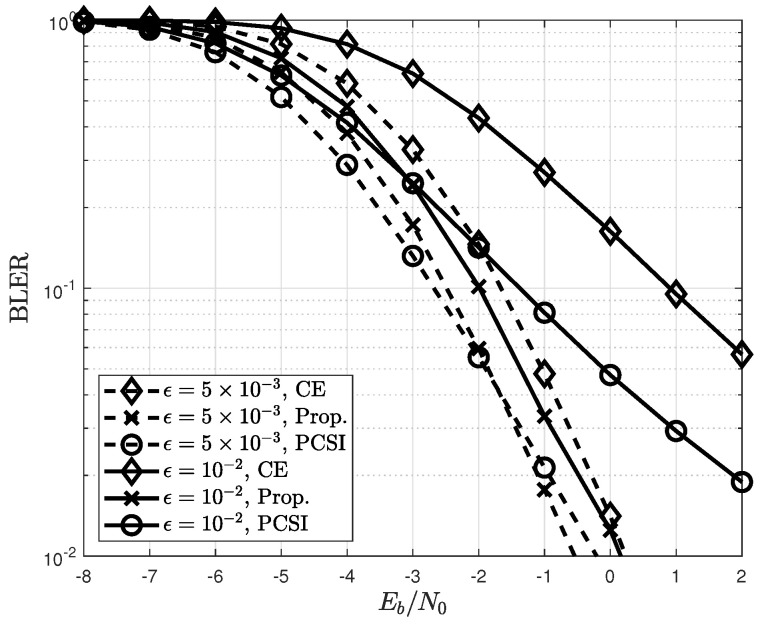
BLERs of the proposed channel estimators in time-varying channels.

Figure 9 and Figure 10 are the results obtained using the proposed channel estimator in time-varying channels. Specifically, a first-order Gaussian–Markov process used in [29,30] was adopted.In this process, the channel matrix at time slot *n* is defined as

(31) $$\begin{array}{cc} H^{\left(n\right)} & =\sqrt{1-\epsilon^{2}}H^{\left(n-1\right)}+\epsilon e^{\left(n\right)}, \end{array}$$

where $n\in N_{b,d}$ for $b\in \{1,2,\ldots ,N_{b}\}$ and $d\in \{1,2,\ldots ,N_{d}\}$. ϵ∈[0,1] is a temporal correlation coefficient depending on the velocity, and $H^{\left(0\right)}$ is an initial channel estimate. Each element in $e^{\left(n\right)}\in C^{N_{r}\times N_{t}}$ is assumed to follow CN(0,1). Temporal correlation coefficients $\epsilon =5\times 10^{-3}$ and $\epsilon =10^{-2}$ are used for the simulations.

Figure 9 shows the variation in the NMSE of the proposed channel estimator with the discounting factor. When a channel varies over time as $\epsilon =5\times 10^{-3}$, an NMSE with γ=0.1 is better than it is with γ=0.9. This is because the rewards at the future states in the time-varying channels are insignificant; therefore, a small value of the discounting factor is preferable. By contrast, when the channels are time-invariant, the rewards at the future states as well as those at the current state are important. Thus, the large value of γ=0.9 improves the NMSE compared to γ=0.1. Figure 10 compares the BLERs of the proposed and conventional channel estimators. When $\epsilon =10^{-2}$, the BLERs of the CE are severely degraded because the CE method cannot capture the channel variation. However, the proposed channel estimator shows robustness in time-varying channels because the channel variation can be tracked efficiently by selecting the detected symbols.

## 6. Conclusions

In this paper, a low-complexity algorithm for an RL-based channel estimator for MIMO systems was proposed. The proposed channel estimator adaptively selects detected symbols as additional pilot symbols to minimize the channel estimation error. In this study, an MDP problem was introduced, and a practical algorithm to solve it was developed using backup samples and data subblocks. Simulation results showed that the proposed channel estimator significantly improves the BLER and the NMSE compared to the conventional channel estimator.

A future direction of this study is to develop the RL approach for a realistic channel. The proposed method was derived based on the Rayleigh fading channel, but the realistic channel may have a line of sight. Thus, the MDP under the Rician fading channel should be investigated. Another important direction is to develop the RL approach for frequency-selective channels. In frequency-selective channels, the use of multiple sub-carriers can increase computational complexity considerably. Thus, a low-complexity algorithm in frequency-selective channels is necessary. Lastly, the RL approach can also be extended to other advanced channel estimators, such as the iterative method. In this method, the MDP should be reformulated according to the channel estimator.

## Data Availability

Not applicable.

## Appendix A. Proof of Theorem 1

Although the basic derivation of the optimal policy is based on [26], two additional factors are considered, which are presented in this appendix. The first is that the proposed derivation considers a discounting factor in the Q-value; thus, the intermediate rewards do not disappear, unlike in [26]. Second, a finite number of backup samples are used in the derivation; thus, the rewards that do not exploit the APPs are approximated differently compared to [26].

Under the assumption that the discounting factor is 1, the future value function at state ${\tilde{U}}_{n+N+1}^{\left(a,j\right)}\left(S_{n}\right)\in S_{n+N+1}$ is expressed by substituting (14) in (28), as follows:

(A1) $$\begin{array}{cc} V^{★}\left({\tilde{U}}_{n+N+1}^{\left(a,j\right)}\left(S_{n}\right)\right)\; & =\mathrm{Tr}[C_{e}\left({\tilde{U}}_{n+N+1}^{\left(a,j\right)}\left(S_{n}\right)\right)-C_{e}\left({\hat{U}}_{n+N+2}^{\left(a,j\right)}\left(S_{n}\right)\right)\\ \; & +\sum_{m=n+N+2}^{N_{b,d}\left(T_{b}\right)}C_{e}\left({\hat{U}}_{m}^{\left(a,j\right)}\left(S_{n}\right)\right)-C_{e}\left({\hat{U}}_{m+1}^{\left(a,j\right)}\left(S_{n}\right)\right)]\\ \; & =\mathrm{Tr}\left[C_{e}\left({\tilde{U}}_{n+N+1}^{\left(a,j\right)}\left(S_{n}\right)\right)-C_{e}\left({\hat{U}}_{N_{b,d}\left(T_{b}\right)+1}^{\left(a,j\right)}\left(S_{n}\right)\right)\right]. \end{array}$$

By substituting (14) and (A1) into (27), the Q-value function can be obtained as follows:

(A2) $$\begin{array}{cc} Q\left(S_{n},a\right) & =\sum_{j\in J_{a}}T_{n+1}^{\left(a,j\right)}\left(S_{n}\right)\mathrm{Tr}\left[C_{e}\left(S_{n}\right)+\sum_{m=n}^{n+N}\gamma^{m-n}\left(\gamma -1\right)C_{e}\left({\tilde{U}}_{m+1}^{\left(a,j\right)}\left(S_{n}\right)\right)-\gamma^{N+1}C_{e}\left({\hat{U}}_{N_{b,d}\left(T_{b}\right)+1}^{\left(a,j\right)}\left(S_{n}\right)\right)\right]. \end{array}$$

Thus, the optimal policy in (17) is expressed as

(A3) $$\begin{array}{cc} \pi^{★}\left(S_{n}\right)\; & =\underset{a\in \{0,1\}}{\mathrm{argmax}}Q\left(S_{n},a\right)\\ \; & =I\left[\left(Q\left(S_{n},1\right)-Q\left(S_{n},0\right)\right)\geq 0\right]\\ \; & =I[\mathrm{Tr}[\sum_{m=n}^{n+N}\gamma^{m-n}\left(\gamma -1\right)\left(\sum_{j=1}^{K}\theta_{j}\left[n\right]C_{e}\left({\tilde{U}}_{m+1}^{\left(1,j\right)}\left(S_{n}\right)\right)-C_{e}\left({\tilde{U}}_{m+1}^{\left(0,0\right)}\left(S_{n}\right)\right)\right)\\ \; & -\gamma^{N+1}\left(\sum_{j=1}^{K}\theta_{j}\left[n\right]C_{e}\left({\hat{U}}_{N_{b,d}\left(T_{b}\right)+1}^{\left(1,j\right)}\left(S_{n}\right)\right)-C_{e}\left({\hat{U}}_{N_{b,d}\left(T_{b}\right)+1}^{\left(0,0\right)}\left(S_{n}\right)\right)\right)]\geq 0]. \end{array}$$

In (17), the optimal policy is determined by the difference between the error covariance matrices with a=0 and a=1. The error covariance matrices for virtual states ${\tilde{U}}_{m}^{\left(a,j\right)}\left(S_{n}\right)$ and ${\hat{U}}_{m}^{\left(a,j\right)}\left(S_{n}\right)$ are derived as described below.

### Appendix A.1. Error Covariance Calculation for ${\tilde{U}}_{m}^{\left(a,j\right)}\left(S_{n}\right)$

To obtain the error covariance matrix, the distribution of the received symbols, ${\bar{y}}_{r}^{H}\left({\tilde{U}}_{m}^{\left(a,j\right)}\left(S_{n}\right)\right)$, in (2) is required, which is given by

(A4) $${\bar{y}}_{r}^{H}\left({\tilde{U}}_{m}^{\left(a,j\right)}\left(S_{n}\right)\right)∼\mathrm{CN}\left(0_{|M_{m}^{\left(a\right)}|},{\left(X_{m}^{\left(a,j\right)}\right)}^{H}X_{m}^{\left(a,j\right)}+N_{0}I_{|M_{m}^{\left(a\right)}|}\right),$$

for $j\in J_{a}$ and a∈A. Thus, the error covariance matrix in (A3) is computed using the result in [26], as follows:

(A5) $$\begin{array}{cc} C_{e}\left({\tilde{U}}_{m}^{\left(a,j\right)}\left(S_{n}\right)\right) & =N_{0}Q_{m}^{\left(a\right)}-N_{0}^{2}{\left(Q_{m}^{\left(a\right)}\right)}^{2}+Q_{m}^{\left(a\right)}D_{m}^{\left(a,j\right)}{\left(D_{m}^{\left(a,j\right)}\right)}^{H}Q_{m}^{\left(a\right)}, \end{array}$$

where

$$\begin{array}{cc} Q_{m}^{\left(a\right)} & ={\left({\hat{X}}_{m}^{\left(a\right)}{\left({\hat{X}}_{m}^{\left(a\right)}\right)}^{H}+N_{0}I_{N_{t}}\right)}^{-1}\overset{\left(a\right)}{=}\left\{\begin{array}{cc} {\left({\hat{X}}_{n}{\hat{X}}_{n}^{H}+\sum_{l=n+1}^{m-1}\tilde{x}\left[l\right]{\tilde{x}}^{H}\left[l\right]+N_{0}I_{N_{t}}\right)}^{-1}, & a=0,\\ {\left({\left(Q_{m}^{\left(0\right)}\right)}^{-1}+\hat{x}\left[n\right]{\hat{x}}^{H}\left[n\right]\right)}^{-1}, & a=1. \end{array}\right.\\ D_{m}^{\left(a,j\right)} & ={\hat{X}}_{m}^{\left(a\right)}{\left({\hat{X}}_{m}^{\left(a\right)}-X_{m}^{\left(a,j\right)}\right)}^{H}+N_{0}I_{N_{t}}\\ \; & \overset{\left(b\right)}{=}\left\{\begin{array}{cc} {\hat{X}}_{n}{\left({\hat{X}}_{n}-X_{n}\right)}^{H}+\sum_{l=n+1}^{m-1}\hat{x}\left[l\right]{\left(\hat{x}\left[l\right]-\tilde{x}\left[l\right]\right)}^{H}+N_{0}I_{N_{t}}, & j\in J_{a},a=0,\\ D_{m}^{\left(0,0\right)}+\hat{x}\left[n\right]{\left(\hat{x}\left[n\right]-x_{j}\right)}^{H}, & j\in J_{a},a=1. \end{array}\right. \end{array}$$

Thus, the matrix $Q_{m}^{\left(1\right)}$ is re-expressed as

(A6) $$\begin{array}{cc} Q_{m}^{\left(1\right)} & =Q_{m}^{\left(0\right)}-\frac{Q_{m}^{\left(0\right)}\hat{x}\left[n\right]{\hat{x}}^{H}\left[n\right]Q_{m}^{\left(0\right)}}{1+{\hat{x}}^{H}\left[n\right]Q_{m}^{\left(0\right)}\hat{x}\left[n\right]}. \end{array}$$

In addition, $D_{m}^{\left(1,j\right)}{\left(D_{m}^{\left(1,j\right)}\right)}^{H}$ can be computed as

(A7) $$D_{m}^{\left(1,j\right)}{\left(D_{m}^{\left(1,j\right)}\right)}^{H}=\left(D_{m}^{\left(0,0\right)}+{\hat{d}}_{n}\right){\left(D_{m}^{\left(0,0\right)}+{\hat{d}}_{n}\right)}^{H}+{\hat{\delta }}_{n}\hat{x}\left[n\right]{\hat{x}}^{H}\left[n\right],$$

where ${\hat{d}}_{n}=\hat{x}\left[n\right]{\left(\hat{x}\left[n\right]-\tilde{x}\left[n\right]\right)}^{H}$, and

(A8) $$\begin{array}{cc} {\hat{\delta }}_{n} & =\sum_{j=1}^{K}\theta_{j}\left[n\right]∥\hat{x}\left[n\right]-x_{j}{∥}^{2}-{∥\hat{x}\left[n\right]-\tilde{x}\left[n\right]∥}^{2}. \end{array}$$

### Appendix A.2. Error Covariance Calculation for ${\hat{U}}_{m}^{\left(a,j\right)}\left(S_{n}\right)$

Similar to the description in Appendix A.1, the error covariance matrix for ${\hat{U}}_{m}^{\left(a,j\right)}\left(S_{n}\right)$ can be obtained as

(A9) $$\begin{array}{cc} C_{e}\left({\hat{U}}_{N_{b,d}\left(T_{b}\right)+1}^{\left(a,j\right)}\left(S_{n}\right)\right) & =N_{0}Q_{N_{b,d}\left(T_{b}\right)+1}^{\left(a\right)}-N_{0}^{2}{\left(Q_{N_{b,d}\left(T_{b}\right)+1}^{\left(a\right)}\right)}^{2}\\ & +Q_{N_{b,d}\left(T_{b}\right)+1}^{\left(a\right)}D_{N_{b,d}\left(T_{b}\right)+1}^{\left(a,j\right)}{\left(D_{N_{b,d}\left(T_{b}\right)+1}^{\left(a,j\right)}\right)}^{H}Q_{N_{b,d}\left(T_{b}\right)+1}^{\left(a\right)}, \end{array}$$

where $Q_{N_{b,d}\left(T_{b}\right)+1}^{\left(0\right)}$ and $D_{N_{b,d}\left(T_{b}\right)+1}^{\left(0,0\right)}$ can be obtained from (26) as

$$\begin{array}{cc} Q_{N_{b,d}\left(T_{b}\right)+1}^{\left(0\right)} & ={\left({\hat{X}}_{n}{\hat{X}}_{n}^{H}+\sum_{l=n+1}^{n+N}\tilde{x}\left[l\right]{\tilde{x}}^{H}\left[l\right]+\sum_{l=n+N+1}^{N_{b,d}\left(T_{B}\right)}\hat{x}\left[l\right]{\hat{x}}^{H}\left[l\right]+N_{0}I_{N_{t}}\right)}^{-1}\\ D_{N_{b,d}\left(T_{b}\right)+1}^{\left(0,0\right)} & =D_{n+N+1}^{\left(0,0\right)}. \end{array}$$

To resolve the detected symbols after n+N+1 in (A9), $Q_{N_{b,d}\left(T_{b}\right)+1}^{\left(0\right)}$ is further approximated. To this end, the expectation value of $Q_{N_{b,d}\left(T_{b}\right)+1}^{\left(0\right)}$ is used with Jensen’s inequality in (A9), yielding

(A10) $$\begin{array}{cc} Q_{N_{b,d}\left(T_{b}\right)+1}^{\left(0\right)} & \approx E\left\{{\left({\hat{X}}_{n}{\hat{X}}_{n}^{H}+\sum_{l=n+1}^{n+N}\tilde{x}\left[l\right]{\tilde{x}}^{H}\left[l\right]+\sum_{l=n+N+1}^{N_{b,d}\left(T_{b}\right)}\hat{x}\left[l\right]{\hat{x}}^{H}\left[l\right]+N_{0}I_{N_{t}}\right)}^{-1}\right\}\\ \; & \geq {\left({\hat{X}}_{n}{\hat{X}}_{n}^{H}+\sum_{l=n+1}^{n+N}\tilde{x}\left[l\right]{\tilde{x}}^{H}\left[l\right]+\left(N_{b,d}\left(T_{b}\right)-\left(n+N-1\right)+N_{0}\right)I_{N_{t}}\right)}^{-1}, \end{array}$$

where $E\left\{\hat{x}\left[n\right]{\hat{x}}^{H}\left[n\right]\right\}\approx E\left\{x\left[n\right]x^{H}\left[n\right]\right\}=I_{N_{t}}$. Thus, by substituting (A5) and (A9) into (A3), a result in (29) is obtained where $Q_{m}=Q_{m+1}^{\left(0\right)}$ and $D_{m}=D_{m+1}^{\left(0,0\right)}$.
